# Statin use and association with colorectal cancer survival and risk: case control study with prescription data linkage

**DOI:** 10.1186/1471-2407-12-487

**Published:** 2012-10-22

**Authors:** Fatim Lakha, Evropi Theodoratou, Susan M Farrington, Albert Tenesa, Roseanne Cetnarskyj, Farhat V N Din, Mary E Porteous, Malcolm G Dunlop, Harry Campbell

**Affiliations:** 1 Centre for Population Health Sciences, University of Edinburgh, Teviot Place, Edinburgh, EH8 9AG, UK; 2 Colon Cancer Genetics Group, Western General Hospital, University of Edinburgh, Crewe Road, Edinburgh, UK; 3 School of Nursing, Midwifery & Social Care, Faculty of Health, Life and Social Sciences, Edinburgh Napier University, Edinburgh, UK; 4 South East Scotland Genetic Service, Western General Hospital, Edinburgh, UK

## Abstract

**Background:**

In Scotland colorectal cancer (CRC) is the third most common cancer and a leading cause of cancer death. Epidemiological studies have reported conflicting associations between statins and CRC risk and there is one published report of the association between statins and CRC survival.

**Methods:**

Analysis was carried out on 309 cases and 294 controls from the Scottish Study of Colorectal Cancer (SOCCS). Cox’s hazard and logistic regression models were applied to investigate the association between statin use and CRC risk and survival.

**Results:**

In an adjusted logistic regression model, statins were found to show a statistically significant association for three of the four statin variables and were found to not show a statistically significant association with either all-cause or CRC-specific mortality (OR 0.49; 95%CI 0.49-1.36; p-value = 0.17 and OR 0.33; 95%CI 0.08-1.35; P-value = 0.12, respectively).

**Conclusion:**

We did find a statistically significant association between statin intake and CRC risk but not statin intake and CRC-specific mortality. However, the study was insufficiently powered and larger scale studies may be advisable.

## Background

Scotland has one of the highest incidences of colorectal cancer in the world. Colorectal cancer (CRC) is the third most commonly diagnosed cancer in both men and women (15% of cancer cases in men; 11.6% of cancer cases in women). Approximately 3,900 new cases are diagnosed each year and 95% of cases occur in people aged over 50 years. Over recent years both the incidence and mortality rates have fallen for both sexes. However, CRC remains the second most common cause of cancer deaths for men (10.1% of cancer-related deaths) and the third for women (9.6% of cancer related deaths) with approximately 1500 people dying of the disease in Scotland each year [1].

The main risk factors, excluding genetic, for colorectal cancer are dietary, obesity, lack of physical activity and smoking. The prevalence of each of these risk factors is also high in the Scottish population. Additionally, Scotland’s overall health is comparatively poor for a Western county, particularly amongst people of working age. This includes heart disease.

Statins, also known as 3-hydroxy-3-methylglutaryl coenzyme A (HMG-CoA) reductase inhibitors, were first prescribed in Scotland in 1989. They have revolutionised the treatment of hypercholesterolaemia [2], by lowering serum cholesterol and reducing cardiac morbidity and mortality in both primary and secondary prevention of coronary artery disease [2,3]. There has been a consistent increase in prescribing of statins, which reflects the increase in prescribing of drugs for cardiovascular disease (Additional file 1: Supplementary material 1).

Their beneficial effects are usually attributed to their capacity to reduce endogenous cholesterol synthesis [4,5]. They competitively inhibit HMG-CoA reductase, the major rate-limiting enzyme that controls the conversion of HMG-CoA to mevalonic acid (MA) [6,7]. These mevalonate-derived prenyl groups enable precise cellular localisation and function of many proteins involved in important intracellular signalling pathways (e.g. Ras and Rho proteins) [6,7]. Therefore, besides lowering cholesterol levels, statins exert effects on many essential cellular functions including cell proliferation, differentiation, and survival as well as participating in the regulation of cell shape and motility [8]. It is these other effects of MA and the fact that many malignant cells present an increased HMG-CoA reductase activity, which suggest that selective inhibition of the HMG-CoA reductase enzyme could lead to a new chemotherapy for cancer disease [9].

Results obtained *in vitro* have demonstrated that statins possess a number of anti-tumour effects and through a variety of potential mechanisms (Additional file 1: Supplementary material 2). *In vivo* studies have, in the main, endorsed *in vitro* results by the display of antitumour effects in numerous animal tumour models resulting in retardation of tumour growth; inhibition of angiogenesis and/or inhibition of the metastatic process [10-15]. Additionally a number of studies have legitimised the potential of statins to significantly increase the chemopreventive efficacy of other anti-tumour treatments at doses much lower that are needed for their anti-proliferative effects [16-21].

Epidemiological studies have in the main concentrated on the association with risk of colorectal cancer. Results of these have been inconsistent (Additional file 1: Supplementary material 3 and 4) and the exact role of statins remains to be elucidated. More recently there has been a growing interest in the association of statins with disease progression and survival. The former has been explored in only two studies [22,23] and the latter in one [23]. Findings from these indicated that long term use of statins may be associated with a less advanced tumour stage and a better survival rate [22,23].

The objective, addressed by this study, was to explore the association between statin use and colorectal cancer risk, progression and survival in a Scottish population. To date no study has investigated these associations of statin use and CRC in a Scottish population and data otherwise remains inconclusive.

## Methods

### Ethics statement

Ethical approval was obtained from the Multi Centre Research Ethics committee for Scotland (MREC) and relevant Local Research Ethics committees. All participants provided written informed consent.

### Study population

The study population comprised a subpopulation of cases and controls resident in Tayside (309 cases and 294 controls) who were involved in the Scottish Study of Colorectal Cancer (SOCCS; original sample size: 3,455 cases and 3,396 controls).

### SOCCS study

The aim of the SOCCS study was to investigate the genetic, diet and lifestyle factors which influence colorectal carcinogenesis. Incident cases of adeno-carcinoma of the colon or rectum in patients aged 16–79 presenting to surgical units in Scottish hospitals between 1999 and 2006 were prospectively recruited into the SOCCS study. Research staff were based in each of the main surgical centres throughout Scotland. This minimised ascertainment bias and assured recruitment within four weeks of admission thus limiting survival bias due to rapid attrition of cancer-related deaths. Those not approached were: patients who died before ascertainment; patients too ill to participate; patients with a recurrence of CRC or patients who were unable to give informed consent. 32% of all ascertained incident cases of CRC were recruited to participate in SOCCS. Matched controls (on age (±1 year), sex and region of residence) were identified at random from a population-based register (community health index) and invited via their general practitioner to participate. Participation rates among those approached were approximately 52% for cases and 39% for controls. Both the food frequency questionnaire and the lifestyle and cancer questionnaire had to be completed to a sufficiently high level for analysis to be valid. Thus valid analysis was only possible for 68% of recruited cases and 88% of recruited controls (see Theodoratou E et al., 2007 [24] for further recruitment details). For the purposes of this particular study data linkage was only feasible for those resident in Tayside, Scotland and this further reduced the total sample size. (See Additional file 1: Supplementary material 5 for flow sheet).

### Lifestyle and dietary data

Subjects completed one questionnaire about their general lifestyle and one semi-quantitative food frequency questionnaire (Scottish Collaborative Group FFQ, Version 6.41; http://www.foodfrequency.org). The main characteristics of these questionnaires and data on FFQ validity have been previously described [24-26].

### Survival analysis data

Up to the censoring date (31/08/2009), there were 106 deaths in the 309 cases that were included in the current analysis. Cause of death was determined by examining all death certificates in a blinded manner with respect to statin use. Ninety-one of the 106 deaths were due to CRC (84% of all deaths). Each recruited cancer subject was assigned an American Joint Committee on Cancer (AJCC) stage derived from a synthesis of clinical, pathological and imaging information (Additional file 1: Supplementary material 6). Staging involved contact with individual patient general practitioners and surgeons, radiology and pathology departments, as well as each of the managed clinical networks throughout Scotland.

### Statin data

Dispensed prescription data (medication, quantity, the pharmacy and the prescriber) is routinely collected for the purposes of fee payment to pharmacies. The Health Informatics Centre (HIC) in Dundee, Tayside has reliably collected these data together with the CHI (Community Health Index) numbers from all Tayside community dispensed prescriptions. CHI is a population register, which is used in Scotland for healthcare purposes. The CHI number uniquely identifies a person on the index.

Data linkage was undertaken with the assistance of the HIC, which provided the CHI numbers and statin data, and the Information Statistics Division of National Services (ISD) which provided the CHI numbers for all our study participants (Additional file 1: Supplementary material 7).

Statin use was described using four different variables: one or more prescriptions dispensed at least two months pre-recruitment; one or more prescriptions dispensed at least seven months pre-recruitment; two or more prescriptions dispensed at least two months pre-recruitment; and two or more prescriptions dispensed at least seven months pre-recruitment. In the survival analysis statin use was explored as one or more prescriptions dispensed pre-diagnosis, two or more prescriptions dispensed pre-diagnosis and similarly for post-diagnosis.

These variables were chosen for two reasons. Firstly, by looking at those who had at least one statin prescription dispensed we can investigate the total group of statin users. However they may not have taken the tablets from that first prescription and may not have returned for a further prescription. Thus by looking at those who were dispensed two or more prescriptions there is greater likelihood that the medication was indeed used. Secondly, according to the hypothesized underlying biologic mechanism, a minimum exposure period is required for statins to have any effect on the development of cancer. Therefore, statin use was described in two ways, two months pre-recruitment and seven months pre-recruitment. The latter allowed a threshold of at least six months even accounting for the maximal period of time from diagnosis to recruitment in the case of CRC patients.

### Statistical analysis

Data were analysed using SPSS version 14.0 and 19.0 (SPSS Inc. Chicago, IL) and STATA version 10.1 (Stata corp, college station, Texas).

The frequencies and distribution of each variable were checked. Any variable with a skewed distribution was normalised by log transformation. The Pearson *χ*^2^ test and the *t*-test were used to test the difference between cases and controls in terms of categorical and continuous lifestyle and demographic variables. Characteristics of control statin users (≥1 prescription dispensed at least two months pre-recruitment) and control non-users were compared in an identical manner to above.

Endpoints investigated were differences in risk (incidence) of colorectal cancer between statin users and non-users, and differences in staging and in mortality from colorectal cancer between statin users and non-users.

Conditional logistic regression models were used in risk analysis and Cox’s hazard models were used for survival in estimating the strength of the association between CRC and statin use for each of the statin categories. Logrank tests and Cox’s hazard models (crude and adjusted for stage of cancer, age and sex) estimated statin effects on all-cause and CRC-specific mortality. For each statin category the model was adjusted for matching factors (age +/−1 year, sex and region of residence); family history of CRC (low and medium/high); past medical history of cancer, past medical history of bowel disease (including irritable bowel syndrome), body mass index (BMI) (kg/m^2^ continuously), smoking (3 categories – current, former and never), physical activity (hours of cycling/sport per week) and regular NSAID intake (yes versus no).

## Results

### SOCCS study participation

52% of colorectal cancer cases, and 39% of controls, approached agreed to participate. Analysis of those who participated to those who did not found that the two groups were statistically significantly different for age, sex and health board area of residence and deprivation score (Additional file 1: Supplementary material 8–11).

### SOCCS study and statin use

Over 99% of the 309 cases and 294 controls studied were Caucasian, 53% were male and 50% were 63 years old or older.

There were no significant differences between cases and controls in terms of age, sex, smoking status, physical activity, alcohol intake, energy intake, deprivation category, past medical history of bowel disease, regular use of NSAIDs and hormone replacement therapy or hormonal contraception (among women) (Table 1). There was a significant difference in BMI (OR 3.84; 95%CI 1.27, 12.5; p-value = 0.02). Cases were also significantly more likely to have a personal history of cancer (OR 2.13; 95%CI 1.05, 4.17; P-value = 0.03) and/or a family history of CRC (OR 71.6; 95% CI 9.84, 520.1; P-value < 0.001) (Table 1).

**Table 1:** Demographic characteristics and lifestyle factors of the study population

**Variables**	Cases (n = 309)*	Controls (n = 294)*	**P-value †**
Age at recruitment	60.0 (11.8)	61.4 (13.96)	0.19
Sex:			
Men	160 (51.8)	161 (54.8)	
Women	149 (48.2)	133 (45.2)	0.46
FH risk***:			
Low	226 (78.7)	265 (99.6)	
Medium/High	61 (21.3)	1 (0.4)	<0.001
Smoking status:			
Never	103 (33.3)	90 (30.6)	
Former	87 (28.2)	115 (39.4)	
Current	41 (13.3)	45 (15.3)	0.18
Not known	78 (25.2)	44 (15.0)	
Physical activity (cycling & other sport in hours/week) ‡			
0	125 (56.6)	128 (53.6)	
0.1-3.5	56 (25.3)	65 (27.2)	
3.6-7	23 (10.4)	24 (10.0)	
>7	17 (7.7)	22 (9.2)	0.48
BMI‡ ¥:			
<25	92 (29.8)	80 (27.2)	
25-29.9	102 (33.0)	105 (35.7)	
≥30	36 (11.7)	64 (21.8)	0.02
Unknown	79 (25.6)	45 (15.3)	
Alcohol intake (g/day)‡	12.7 (14.6)	12.9 (13.9)	0.76
Energy intake (kJ/day)‡	11016 (3896)	11054 (4572)	0.80
DEPCAT^††^			
1	33 (10.7)	33 (11.2)	
2	76 (24.6)	66 (22.4)	
3	78 (25.2)	79 (26.9)	
4	69 (22.3)	66 (22.4)	
5	24 (7.8)	22 (7.5)	
6	27 (8.7)	27 (9.2)	
7	2 (0.6)	1 (0.3)	0.99
PMH Bowel disease (incl. IBS)	18 (7.8)	23 (9.3)	0.56
PMH Cancer ±	24 (10.3)	13 (5.1)	0.03
Statin use: at least 1	25 (8.1)	44 (15.0)	<0.01
prescription dispensed 2/12	Male: 18	Male: 31	Male: <0.05
pre-recruitment	Female: 7	Female: 13	Female:0.10
Statin use: at least	24 (7.8)	38 (12.9)	0.04
1prescription dispensed 7/12	Male: 17	Male: 28	Male: 0.08
pre-recruitment	Female: 7	Female: 10	Female: 0.32
Statin use: 2+ prescriptions	24 (7.8)	38 (12.9)	0.04
dispensed. First being at least	Male: 17	Male: 27	Male: 0.11
2/12 pre-recruitment	Female: 7	Female: 11	Female: 0.22
Statin use: 2+ prescriptions	23 (7.4)	34 (11.6)	0.084
dispensed. First prescription at	Male: 16	Male: 25	Male: 0.14
least 7/12 pre-recruitement	Female: 7	Female: 9	Female: 0.45
Regular use of NSAIDs**:			
Yes	53 (69.7)	87 (70.7)	
No	165 (17.1)	146 (17.9)	0.003
Not known	91 (13.1)	61 (11.4)	
HRT use:			
Yes	30 (28.0)	40 (37.0)	
No	76 (71.0)	68 (63.0)	0.17
Not known	1 (0.9)	0 (0)	
Hormonal contraception use:			
Yes	36 (33.6)	40 (37.0)	
No	70 (65.4)	67 (62.0)	0.60
Not known	1 (0.9)	1 (0.9)	

* Mean values and in parenthese standard deviations for quantitative variables; number of subjects and in parentheses percentages for categorical variables.

† P-values from the Pearson *χ*^2^ for categorical variables; from *t*-test for continuous variables. All statistical tests were 2-sided.

‡ P-values were computed from the natural logarithmic transformed variables.

** Regular use = at least four times a week for at least one month.

†† DEPCAT (Carstairs deprivation index) based on the 2001 Census data; 7 categories ranging from very low deprivation (DEPCAT 1) to very high deprivation (DEPCAT 7).

¥ In calculating the BMI the weight and height used were from 1 year before diagnosis for cases and one year before recruitment for controls.

± Information on past cancers was self-reported by patients via the lifestyle questionnaire. The question that was asked was: “up until a year ago had you ever been given a diagnosis of cancer?”. Type and staging were not requested.

*** Family history risk was assigned according to the Scottish guidelines: According to the Scottish Executive cancer guidelines ( http://www.sehd.scot.nhs.uk/), the criteria for high family history risk of colorectal cancer are: 1) at least three family members affected by colorectal cancer or at least two with colorectal cancer and one with endometrial cancer in at least two generations; one affected relative must be ≤50 years old at diagnosis and one of the relatives must be a first degree relative of the other two; or 2) presence of the HNPCC syndrome; or 3) untested first degree relatives of known gene carriers. The criteria for moderate risk are: 1) one first degree relative affected by colorectal cancer when aged <45 years old; or 2) two affected first degree relatives with one aged <55 years old; or 3) three affected relatives with colorectal or endometrial cancer, who are first degree relatives of each other and one a first degree relative of the consultant. Individuals that do not fulfil all the above criteria are classified as low family history risk (Scottish Executive cancer guidelines).

Age, sex and BMI were statistically significantly different between statin users, (those who had dispensed at least one prescription two months before recruitment) and non-users (Additional file 1: Supplementary material 12). When men and women were explored separately there were no significant differences between users and non-users in women (Additional file 1: Supplementary material 13). However in men there was a small but significant difference in age between statin users and non-users (Additional file 1: Supplementary material 14). Use of sigmoidoscopy and/or colonoscopy was explored and no association was found between statin users and non users amongst anyof the different statin groups when missing data were excluded (Additional file 1: Supplementary material 15).

### Statins and risk of colorectal cancer

Table 2 presents the results of the logistic regression models looking at the relationship between CRC and each of the four variables of statin use. Statin use was associated with a statistically significantly reduced risk of CRC for one of the four variables in the unadjusted model; OR = 0.52 95% CI = 0.31, 0.89 for those who had at least one prescription dispensed at least two months pre-recruitment (Table 2). However after adjusting for confounding factors, the association was significant for three variables; OR = 0.33 95% CI 0.15, 0.69 for those who had at least one prescription dispensed at least two months pre-recruitment; OR = 0.39 95% CI 0.18, 0.85 for those who had at least one prescription dispensed seven months pre-recruitment and OR = 0.42 95%CI 0.19, 0.92 for those who had at least two prescriptions dispensed the first being at least two months pre-recruitment.

**Table 2:** Association between colorectal cancer and statin use among 309 cases and 294 control patients (unadjusted model) and 190 cases and 209 control patients in the adjusted model

**Statin use**	**Unadjusted model**			**Adjusted model**		
	**Cases (309)**	**Controls (294)**	Basic OR* (95% CI)	**Cases (194)**	**Controls (211)**	**Adjusted OR† (95% CI)**
			**P-value**			**P-value**
No use of statins	284	250	1.0 (referent)	154	145	1.0 (referent)
≥1dispensed prescription at least 2 months pre-recruitment	25	44	0.52 (0.31, 0.89)	15	38	0.33 (0.15, 0.69)
			0.016 **			0.004**
≥1dispensed prescription at least 7 months pre-recruitment	24	38	0.60 (0.35, 1.04)	14	32	0.39 (0.18, 0.85)
			0.067			0.017**
≥2 dispensed prescriptions at least 2 months pre-recruitment	24	38	0.60 (0.35, 1.03)	14	32	0.42 (0.19, 0.92)
			0.064			0.030**
≥2 dispensed prescriptions at least 7 months pre-recruitment	23	34	0.65 (0.37, 1.14)	13	28	0.49 (0.22, 1.08)
			0.135			0.077

* Adjusted for matching factors (age +/−1 year), sex and region of residence). *OR* = Odds ratio; *CI* = confidence interval.

† Adjusted for matching factors ((age +/−1 year), sex and region of residence), Family history of cancer, past medical history of cancer, past medical history of bowel disease, BMI, smoking, physical activity and regular NSAID intake.

** statistically significant at p < 0.05.

For the logistic regression the sample size was 405 (194 cases of colorectal cancer and 211 controls) due to there being at least one piece of missing data for 198 study participants.

The analysis was repeated including only those with complete data for each of the potential confounders (PMH Cancer, FH of Cancer, PMH IBD, BMI, smoking history, regular NSAID use and physical activity). This reduced the sample size to 405 (194 cases of colorectal cancer and 211 controls). The results were found to be significant for three of the four statin variables in both the unadjusted and adjusted models (Additional file 1: Supplementary material 16).

### Statins, survival and death from colorectal cancer

There were 106 deaths within the group of 309 cases of colorectal cancer. The only significant difference between those deceased and alive was in physical activity (p value 0.008) (Table 3). There was no association found between stage of colorectal cancer at diagnosis and statin use (Additional file 1: Supplementary material 17). Statin use was found to be negatively associated with all-cause mortality and CRC-specific mortality when two or more prescriptions of statin had been dispensed post diagnosis (p-value 0.05 and 0.03; respectively). However, post adjustment for confounding factors this association was no longer significant. None of the other drug categories were found to be associated with either all-cause mortality or colorectal cancer in the unadjusted analysis, when adjusted for AJCC alone and in multivariable analysis (adjusted for age, sex and AJCC) (Table 4). Similarly when physical activity was included in factors adjusted for both alone and in multivariable analysis no association was either all-cause or CRC-specific mortality (data not shown). When only complete data were used then the results were significant for three of the four drug categories both in the unadjusted and adjusted analysis (Additional file 1: Supplementary material 16).

**Table 3:** Demographic characteristics and lifestyle factors of cases (survival analysis)

**Variables**	Deceased (n = 106)*	Alive (n = 202)*	**P-value**
Age at recruitment	60.4 (12.18)	59.8 (11.66)	0.66
Sex:			
Men	56 (52.8)	103 (51.0)	
Women	50 (47.2)	99 (49.0)	0.76
FH risk:			
Low	78 (82.1)	148 (77.1)	
Medium/High	17 (17.9)	44 (22.9)	0.33
Smoking status:			
Never	31 (48.4)	71 (42.8)	
Former	20 (31.3)	67 (40.4)	
Current	13 (20.3)	28 (16.9)	0.44
Physical activity (cycling & other sport in hours/week) ‡			
0	37 (62.7)	88 (54.3)	
0-3.5	6 (10.2)	50 (30.9)	
3.5-7	6 (10.2)	11 (6.8)	
>7	10 (17.0)	13 (8.0)	0.008
BMI‡:			
<25	24 (36.4)	68 (41.7)	
25-29.9	31 (47.0)	71 (43.6)	
≥30	11 (16.7)	24 (14.7)	0.75
Alcohol intake (g/day)‡	13.1	12.6	0.29
	(16.0)	(14.2)	
Energy intake (kJ/day)‡	11222.2	10939.5	0.54
	(3886.9)	(3909.0)	
DEPCAT^††^			
1	11 (10.4)	22 (10.9)	
2	23 (21.7)	53 (26.2)	
3	25 (23.6)	52 (25.7)	
4	29 (27.4)	40 (19.8)	
5	9 (8.5)	15 (7.4)	
6	9 (8.5)	18 (8.9)	
7	0 (0)	2 (1.0)	0.72
PMH Bowel disease (incl. IBS)	6 (9.1)	12 (7.3)	0.65
PMH Cancer	6 (9.1)	17 (10.3)	0.78
Statin use: at least 1 prescription dispensed 2/12 pre-recruitment	8 (7.6)	16 (7.9)	0.91
Statin use: at least 1 prescription dispensed 7/12 pre-recruitment	8 (7.6)	15 (7.4)	0.97
Statin use: 2+ prescriptions dispensed. First being at least 2/12 pre-recruitment	8 (7.6)	15 (7.4)	0.97
Statin use: 2+ prescriptions dispensed. First prescription at least 7/12 pre-recruitement	8 (7.6)	14 (6.9)	0.84
Regular use of NSAIDs**:			
Yes	47 (73.4)	127 (78.4)	
No	17 (26.6)	35 (21.6)	0.43
HRT use:			
Yes	7 (26.9)	23 (28.8)	
No	19 (73.1)	57 (71.3)	0.86
Hormonal contraception use:			
Yes	6 (23.1)	30 (37.5)	
No	20 (76.9)	50 (62.5)	0.18

**Table 4:** Survival analysis for all cause and colorectal cancer mortality by statin intake

**All cause mortality**	***No of events***	***Persons at risk***	***Log rank test***	***Unadjusted Analysis***		***Adjusted for AJCC***		***Multivariable adjusted analysis***	
				**HR**	**95% CI**	**HR**	**95% CI**	**HR**	**95%CI**
Statin use: **at least 1** prescription									
No	91	251		1.00		1.00		1.00	
Yes	15	57	0.11	0.64	0.37, 1.10	0.65	0.37, 1.15	0.58	0.33, 1.03
				P = 0.11		P = 0.14		P = 0.07	
Statin use: **at least 1** prescription dispensed before recruitment									
No	91	251		1.00		1.00		1.00	
Yes	8	26	0.62	0.83	0.40, 1.71	0.76	0.32, 1.38	0.59	0.28, 1.24
				P = 0.62		P = 0.28		P = 0.16	
Statin use: **at least 1** prescription dispensed after recruitment									
No	91	251		1.00		1.00		1.00	
Yes	7	31	0.08	0.51	0.23, 1.09	0.67	0.29, 1.54	0.61	0.26, 1.41
				P = 0.08		P = 0.34		P = 0.24	
Statin use: **2+** prescription									
No	94	259		1.00		1.00		1.00	
Yes	12	49	0.09	0.59	0.33, 1.08	0.63	0.34, 1.15	0.57	0.31, 1.05
				P = 0.09				P = 0.07	
Statin use: **2+** prescription dispensed before recruitment									
No	94	259		1.00		1.00		1.00	
Yes	8	25	0.70	0.87	0.42, 1.79	0.70	0.34, 1.45	0.64	0.31, 1.34
				P = 0.70		P = 0.34		P = 0.24	
Statin use: **2+** prescription dispensed after recruitment									
No	94	259		1.00		1.00		1.00	
Yes	4	24	0.04	0.36	0.13, 0.99	0.54	0.20, 1.49	0.49	0.18, 1.36
				P = 0.05		P = 0.24		P = 0.17	
**Colorectal cancer mortality**				**HR**	**95% CI**	**HR**	**95% CI**	**HR**	**95%CI**
Statin use: at least 1 prescription									
No	79	251		1.00		1.00		1.00	
Yes	12	57	0.09	0.60	0.32, 1.09	0.61	0.32, 1.15	0.56	0.29, 1.07
				P = 0.09		P = 0.13		P = 0.08	
Statin use: at least 1 prescription dispensed before diagnosis									
No	79	251		1.00		1.00		1.00	
Yes	7	26	0.66	0.84	0.39, 1.82	0.65	0.30, 1.42	0.60	0.327, 1.32
				P = 0.66		P = 0.28		P = 0.20	
Statin use: at least 1 prescription dispensed after									
No	79	251		1.00		1.00		1.00	
Yes	5	31	0.05	0.42	0.17, 1.05	0.58	0.21, 1.60	0.54	0.19, 1.50
				P = 0.06		P = 0.29			
Statin use: **2+** prescription									
No	82	259		1.00		1.00		1.00	
Yes	9	49	0.06	0.52	0.26, 1.03	0.56	0.28, 1.11	0.51	0.25, 1.03
				P = 0.06		P = 0.10		P = 0.06	
Statin use: **2+** prescription dispensed before recruitment									
No	82	259		1.00		1.00		1.00	
Yes	7	25	0.73	0.87	0.40, 1.88	0.67	0.31, 1.48	0.64	0.26, 1.40
				P = 0.73		P = 0.33		P = 0.26	
Statin use: **2+** prescription dispensed after recruitment									
No	82	259		1.00		1.00		1.00	
Yes	2	24	0.02	0.21	0.05, 0.86	0.35	0.09, 1.45	0.33	0.08, 1.35
				P = 0.03		P = 0.15		P = 0.12	

## Discussion

### Statins and risk of colorectal cancer

In the univariable analysis of risk a statistically significant protective association between CRC risk and one of the four statin variables was observed. However, after controlling for several potential confounders the association with three of the four statin variables (all but having two or more prescriptions with the first at least seven months pre-recruitment) was statistically significant (Table 1). However the sample size decreased significantly when logistic regression was carried out for the adjusted model and this was reflected in the wide confidence intervals. Thus the results must be treated with caution and larger studies need to be conducted to confirm these findings.

The association between use of statins and colorectal cancer risk has been explored via epidemiological analyses. Whilst the results from some of these studies have supported the hypothesis that statin use may reduce risk of colorectal cancer (Additional file 1: Supplementary material 2, 3), several recent meta-analyses have concluded that there is no association (Additional file 1: Supplementary material 18 and 19). This may not necessarily be the case for a number of reasons. Whilst meta-analysis does provide an explicit systematic approach, in this situation it still has limited sensitivity for detecting carcinogenic potential at a specific cancer site. With respect to the studies included, RCT’s exploring statin use have not been designed to evaluate statins as preventive agents of cancer thus being insufficiently powered, follow-up has been relatively short and external validity, with regard to cancer risk in a post-marketing population of statin users, is questionable as the patients in the trials have been from highly selected groups. Observational studies have also been limited by insufficient numbers, multiple biases including potential misclassification bias and incomplete control of confounding. Hence overall the results remain inconclusive.

### Statins, survival and death from colorectal cancer

Survival analysis did detect an effect on all-cause mortality and CRC-specific mortality in the unadjusted model but did not after adjustment for confounding factors. A post hoc power calculation (Additional file 1: Supplementary material 20) showed that we didn’t have enough power to detect a small survival effect and therefore larger studies are required.

Increasingly the interest in the association between statin use and CRC has been with regard to stage at presentation and survival rate [27]. We found neither a positive nor a negative association with either in our study. To our knowledge only two studies have explored this previously [22,23]. A case–control study by Siddiqui et al. [23] et al. explored, how use of statins might influence presentation of colorectal cancer and survival rate and found that long-term use of statins is associated with a less advanced tumour stage, a higher prevalence of right-sided tumours, a lower frequency of distant metastases, and a better five-year survival rate. This study had a larger sample size than ours. Similarly to the current study, it used a pathology database to identify patients with colorectal cancer thus minimising selection bias. Medical histories were obtained from medical records and did not rely on self-recall thus minimizing recall bias though limiting the data that could be collected and increasing the likelihood of incomplete control of confounding. Statin use was from dispensing data via a pharmacy database, similarly to the current study, thus raising the possibility of misclassification bias.

The second study was a population-based case–control study by Coogan et al. [22]. As part of their exploratory analysis they looked at stage at presentation and found an association between statin use and reduced risk of stage IV cancer at presentation. A strength of this study was that it specifically looked at the association between statin-use with colorectal cancer. It was also the first study where specific attention was paid to statin type, dose and duration of use and where the potentially confounding or synergistic effects of non-steroidal anti-inflammatory drug (NSAID) use were investigated. However, there were several limitations as acknowledged by the authors. There may have been selection bias as participation was voluntary and in the instance of cases physician permission was required before the patient could be approached. However, the associations of CRC with NSAID use, oral contraceptive use and with screening were in the expected direction and thus provided reassurance as to the validity of the data. Recall bias was another potential limitation, since exposure was classified solely on the basis of self-reported drug use with no verification. Accuracy of recall is a known problem in these situations. Similarly to the study by Poynter et al. [28] there was a likelihood of detection bias. A further limitation, as with every observational study, was that of potentially incomplete control of confounding though many efforts were made to control for many potential confounders.

### Strengths and limitations

Our study has several strengths. Firstly active case recruitment within each of the surgical centres throughout Scotland, within 1–3 months of diagnosis, limited both ascertainment and survival bias. Secondly recall bias was minimised by using computerised databases to link dispensed statins to study participants. Thirdly, misclassification bias was minimised by looking at those who had been dispensed at least two prescriptions of statin thus increasing the likelihood of compliance even though drug use was reliant on dispensing data. And fourthly, overestimation was reduced by using a number of statin variables, similarly to Kaye and Jick [29] and Graaf et al. [30], of which two included a six month latent period (plus one month allowance for recruitment.

The main limitation in this study was the lack of availability of prescription data for use in epidemiological research and the reason for the small sample size. Other limitations include that data regarding statin use were only obtainable for 309 cases and 294 controls due to limited availability of data linkage. Also, data were only available from 1996 which potentially may have led to misclassification of some ex-statin users. With statin use only being ascertained from 1996, this is likely to be too short a time-scale as cancer is well known to have a long latency period after exposure to carcinogens [31]. Additionally, whilst efforts were made to reduce survival bias by having research staff based at every surgical centre throughout Scotland those patients who were too ill to participate were excluded as were those who died before ascertainment. Valid analysis of questionnaires, completed by those who consented to participate in the SOCCS study, was only feasible for 68% of cases as compared to 88% of controls. This lower completion rate in cases, as mentioned earlier, is thought to be due to cases being too unwell to fully cooperate and thus inadvertently a further element of survival bias is present in the study as subjects with missing data were excluded from the logistic regression analysis. And there may have been some selection bias since only 32% of incident cases of colorectal cancer were approached. Of those approached only 52% of cases and 39% of controls agreed to participate. When participants were compared to non-participants, for both cases and controls, with respect to age, gender, health board area and deprivation index we found there to be statistically significant differences (Additional file 1: Supplementary material x-y) confirming participation bias. We had very limited data for nonparticipants and were therefore unable to do any further comparisons. Finally our sample size was further reduced, due to missing data (Additional file 1: Supplementary material 21), leaving us with complete data for only 193 cases and 213 controls when we undertook logistic regression (Additional file 1: Supplementary material 16). This further reduced the power of the study.

Given that the use of statins is rapidly increasing worldwide, with more than 10% of the adult population, and 25% of those over 60 years of age in the United States, using statins [32], any association of statins with increased or decreased survival, stage at presentation or risk of colorectal cancer would have a substantial public health impact. Many studies, both trials and observational have been undertaken to date, and it would appear reasonable that rather than expend further resource in repeating such studies, though on a larger scale, it is time to consider exploring a new direction in trying to ascertain if there is a causal link between statin use and CRC. We believe the most plausible option at present is to undertake a meta-analysis of randomised control trials. As mentioned earlier, to date, there have been four meta-analyses of RCTs exploring the colorectal cancer statin link. The latest was in 2007 and whilst there have been numerous RCTs involving statins only six have published their findings with respect to the association with CRC. Possibly by contacting the investigators of each of the RCTs involving statin use it might be possible to ascertain if there is any data available on incidence and mortality of CRC thus allowing both power to be increased as well as exploration of incidence, survival, dose–response, the effects of type and possibly duration of use also.

In conclusion, collective evidence remains inconclusive that statins are protective against colorectal cancer. Whilst laboratory data suggest the biological plausibility of an anti-cancer effect of statins against colorectal cancer, epidemiological data, both when viewed as individual studies and in meta-analyses are inconsistent and not supportive of an impact on risk.

## Competing interests

The authors declare that they have no competing interests.

## Authors' contribution

FL wrote the paper. ET assisted with writing the paper. FL and ET undertook the statistical analysis. SF,AT, RC and FD were responsible for the SOCCS study recruitment, participation and data collection and entry. MP, MD and HC conceived the study and were the principal investigators. All authors read and approved the final manuscript.

## Pre-publication history

The pre-publication history for this paper can be accessed here:

http://www.biomedcentral.com/1471-2407/12/487/prepub

## Supplementary Material

Additional file 1 **Supplementary material 1.** Annual prescribing of statins in Scotland, 2001-6. Supplementary material 2: Potential anti-tumour effects of statins as demonstrated by in-vitro studies. Supplementary material 3. Published cohort studies assessing the association between colorectal cancer and statin use. Supplementary material 4. Published case–control studies to date assessing the association between colorectal cancer and statin use. Supplementary material 5. Flow diagram of recruitment and participation. Supplementaty material 6. General description of AJCC. Supplementary material 7. Mechanism of data linkage via the Health Informatics Centre. Supplementary material 8. Distribution of cases across sex, age, and health board area of residence for participants, non-participants and withdrawn subjects. Supplementary material 9. Reason of no response for non-participants. Supplementary material 10. Distribution of controls across sex, age, health board area of residence and Carstairs deprivation index for participants, non-participants and withdrawn subjects. Supplementary material 11 Carstairs Deprivation Index criteria. Supplementary material 12. Characteristics of statin users versus non-users among control patients. Supplementary material 13. Characteristics of statin users versus non-users among female controls. Supplementary material 14. Characteristics of statin users versus non-users among male controls. Supplementary material 15 Association between statin use and use of sigmoidoscopy and/or colonoscopy. Supplementary material 16 Association between colorectal cancer and statin use among 211 cases and 194 control patients. Supplementary material 17. AJCC distribution according to statin use. Supplementary material 18. Published meta-analyses of RCTs investigating the association between colorectal cancer risk and statin use. Supplementary material 19. Published meta-analyses of observational studies investigating the association between colorectal cancer risk and statin use. Supplementary material 20. Post hoc power calculation. Supplementary material 21. Quantity of missing data for cases and controls. Click here for file
