# Supplementary material for: Statin use and association with colorectal cancer survival and risk: case control study with prescription data linkage
Source: BMC Cancer. 2012 Oct 22;12:487. doi: 10.1186/1471-2407-12-487 (PMC3520719; doi:10.1186/1471-2407-12-487)
Supplement: Additional file 1 — Supplementary material 1. Annual prescribing of statins in Scotland, 2001-6. Supplementary material 2: Potential anti-tumour effects of statins as demonstrated by in-vitro studies. Supplementary material 3. Published cohort studies assessing the association between colorectal cancer and statin use. Supplementary material 4. Published case–control studies to date assessing the association between colorectal cancer and statin use. Supplementary material 5. Flow diagram of recruitment and participation. Supplementaty material 6. General description of AJCC. Supplementary material 7. Mechanism of data linkage via the Health Informatics Centre. Supplementary material 8. Distribution of cases across sex, age, and health board area of residence for participants, non-participants and withdrawn subjects. Supplementary material 9. Reason of no response for non-participants. Supplementary material 10. Distribution of controls across sex, age, health board area of residence and Carstairs deprivation index for participants, non-participants and withdrawn subjects. Supplementary material 11 Carstairs Deprivation Index criteria. Supplementary material 12. Characteristics of statin users versus non-users among control patients. Supplementary material 13. Characteristics of statin users versus non-users among female controls. Supplementary material 14. Characteristics of statin users versus non-users among male controls. Supplementary material 15 Association between statin use and use of sigmoidoscopy and/or colonoscopy. Supplementary material 16 Association between colorectal cancer and statin use among 211 cases and 194 control patients. Supplementary material 17. AJCC distribution according to statin use. Supplementary material 18. Published meta-analyses of RCTs investigating the association between colorectal cancer risk and statin use. Supplementary material 19. Published meta-analyses of observational studies investigating the association between colorectal cancer risk and st [file 1471-2407-12-487-S1.doc]

Supplementary material 1. Annual prescribing of statins in Scotland, 2001-6

Source: ISD website: Prescription cost analysis for Scotland [**http://www.isdscotland.org/isd/info3.jsp?pContentID=2241&p_applic=CCC&p_serv**](http://www.isdscotland.org/isd/info3.jsp?pContentID=2241&p_applic=CCC&p_serv) [accessed 16.05.07]

### Supplementary material 2: Potential anti-tumour effects of statins as demonstrated by in-vitro studies

| Potential effect | Mechanism |
| --- | --- |
| Inhibition of tumour growth by cell cycle arrest | Statins may have growth inhibitory potential. They synchronise tumour cells by blocking transition of G1 to S; G2 to M in the cell cycle or by inducing cell death thereby exerting an antiproliferative effect ^1-8^ |
| Inhibition of tumour growth by induction of apoptosis | Statins may have an impact on both intrinsic and extrinsic pathways as they can upregulate Fas, the receptor for Fas-ligand ^9^, and also induce apoptosis through mitochondrial effects ^10^. The mechanism of HMG-CoA induced apoptosis appears to be mediated predominantly through depletion of geranylgeranylated but not farnesyl pyrophosphate proteins ^6, 11-15^ |
| Inhibition of angiogenesis | At very low (nanomolar) doses statins they display pro-angiogenic activity and at higher (micromolar) doses they display anti-angiogenic activity. ^16^. The anti-angiogenic effects can occur by reducing cytokine-induced production of the major angiogenic mediator vascular endothelial growth factor (VEGF) ^13, 17-19^. In addition, statins can inhibit endothelial cell proliferation and hinder adhesion of endothelial cells to extracellular matrix ^10, 20-22^. |
| Attenuation of metastatic potential | In vitro experiments have shown that statins inhibit cell-signaling pathways associated with the invasive and metastatic properties of cancer ^2, 23^. Underlying mechanisms include prevention of cytokine-induced expression of adhesion molecules such as E-selectin on endothelial cells. ^24^ and inhibition of protease activity; invasion of the basement membrane; cell migration and angiogenesis ^1^. |
| Stimulation of cellular immunity | Statins have been found to reduce the expression of Fas-ligand on tumour cells, a factor known to confer resistance against cellular immunity ^10^. |
| Potentiating the antitumour effects of other agents | Statins have also been found to potentiate the antitumour effects of some cytokines and other chemotherapeutics ^6, 11, 17, 23, 25-33^ |

Supplementary material 3. Published cohort studies assessing the association between colorectal cancer and statin use

| **Study** | **Year** | **Population** | **Cohort size** | **No. CRC cases** | **No. cases in statin users** | **%**  **Male** | **Mean follow-up (yrs)** | **Definition of statin use** | **Statin type** | **Risk All cancers Adjusted RR (95% CI)** | **Risk CRC RR (95% CI)** |
| --- | --- | --- | --- | --- | --- | --- | --- | --- | --- | --- | --- |
| Friis ^34^ | 2005 | Pharmacy database & Danish Cancer Registry analysis (Denmark) | 334,754 | 129 | 55 | 57% statin users; 56% nonstatin lipidlowering drug users; 50% base population | 3.3 | ≥2 prescriptions | All statins | 0.86 (0.78-0.95) | 0.85 (0.65-1.11) |
| Jacobs ^35^ | 2006 | CPS-II Nutrition cohort(USA)  1997-2001 questionnaire-based study | 132,136 | 815 | 183 (current users) 72 (≥5yrs users) | NK | 5 | Current use & ≥5 yrs use | Any lipid lowering agent  (estimated 53% participants using statins) | N/A | Current use: RR=1.03 95%CI=0.85-1.26 ;  ≥5 yrs use: RR=1.09 95%CI=0.83-1.43. |
| Setoguchi ^16^ | 2007 | 65+yr olds statin initiators and glaucoma drug users enrolled in Medicare & drug benefit programs 1994-2003 (Pennsylvania USA) | 24,439 statin users; 7,284 glaucoma drug users | 249 | 190 | 18.2% statin users; 16.4% glaucoma drug users | 2.9 | ≥3 prescriptions filled post initial statin prescription | All statins | N/A | HR=0.96 95%CI=0.70, 1.31 |
| Flick H ^36^ | 2009 | 45-69 yr old men enrolled in a prepaid integrated healthcare maintenance organisation | 69,115 | 171 | 56 | 100% | Max 3.5yrs | Ever use  ≥5yrs estimated | All statins | NA | HR 0.89 (0.61, 1.3)  HR 0.83 (0.43, 1.63) |
| Singh H ^37^ | 2009 | MHHL population registry 010495 to 311295 &>40yrs | 35,739 users; 377,532 neveruse | 6637 | 402 | 51.8% statin users; 46.4% nonusers | Median follow-up 3 years | ≥2 prescrptions | All statins |  | IRR 1.13 95% CI 1.02, 1.25 |

Supplementary material 4. Published case-control studies to date assessing the association between colorectal cancer and statin use

| **Study** | **Year** | **Population** | **No. CRC cases; no. Controls** | **%**  **Male (CRC cases;controls)** | **Mean statin use(yrs)** | **Definition of statin use** | **Statin type** | **All cancers Adjusted OR (95% CI)** | **CRC OR (95% CI)** |
| --- | --- | --- | --- | --- | --- | --- | --- | --- | --- |
| Blais ^38^ | 2000 | Quebec Administrative Health Database | 542 cases; 5420 controls | 44.6: 31.2 | 2.7 | ≥1prescription | Lovastatin;  Pravastatin Simvastatin | 0.72 (0.57-0.92) | Adjusted 0.67 (0.33-1.8) |
| Graaf ^39^ | 2004 | Dutch Database of 8 cities | 3,129 cases; 16,976 controls | 49:49 | 7.2 | ≥1prescription | Pravastatin; Simvastatin; Cerivastatin; Atorvastatin; Fluvastatin | 0.8 (0.66-0.96) | Adjusted 1.07 (0.65-1.74) |
| Kaye ^40^ | 2004 | UK General Practice Database | 3,244 cases; 14,844 controls | 51.1: 50.2 | 6.4 | ≥1prescription | All | 1.0 (0.9-1.2) | Adjusted 0.9 (0.6-1.3) |
| Poynter ^41^ | 2005 | Israeli population-based study 1998-2004 | 1953 cases; 2015 controls | 51.2: 51.2 | Min 5yrs use | >5 yrs of use | All but most commonly Simvastatin & Pravastatin | NA | Unadjusted 0.5 (0.4-0.63);  Adjusted 0.53 (0.38-0.74) |
| Coogan ^42^ | 2007 | Massachusetts population-based study Jan 1^st^ 2001- Nov 30^th^ 2004. | 1809 cases; 1809 controls | 54.8: 54.8 | NK | >3months use and at least 3 times per week | All but most commonly Atorvastatin | NA | Unadjusted 0.82 (0.7-0.95);  Adjusted 0.92 (0.78-1.09) |
| Coogan ^43^ | 2007b | Hospital-based study (NY; Philadelphia; Baltimore)  1991 - 2005 | 4,913 cases;  3900 controls | 47.9: 46.6 | NK | At least 4times per week for ≥3 months commencing ≥1 year pre admission | All types | 1.0 | Adjusted 0.8 (0.5-1.2) |
| Vinogradova ^44^ | 2007 | QReseach database including 454 general practice populations (1995-2005) with >4yrs notes | 5686 cases:  24982 controls | 55.9:56.1 | NK | ≥prescription in 13-48mths prior to index date (i.e. date of diagnosis as noted in database) | Atorvastatin; Cerivastatin; Fluvastatin; Pravastatin; Simvastatin | NA | 0.93 (0.83-1.04) |
| Hoffmeister ^45^ | 2007 | Population based study in Rhine-Neckar-odenwald rgn of SW Germany. ≥30yrs old | 540cases: 614 controls | 58:57 | 6.1yrs | At least 2x/wk for ≥1yr  1-4yrs use  ≥5yrs use | Atorvastatin;  Cerivastatin; Fluvastatin; Lovastatin; Pravastatin; Simvastatin | NA | 0.69 (0.45-1.06)  0.58 (0.33-1.04)  0.71 (0.39-1.28) |
| Farwell ^46^ | 2008 | Veteran Affairs admin & clin database with VA New England VISN-1 pharmaco epidemiology database | 687 cases: 62155 controls | NK | NK | >2prescriptions within 1yr and continued filling of prescriptions for ≥1yr | Atorvastatin; Fluvastatin; Lovastatin; Pravastatin; Simvastatin | 0.74 (0.7-0.78) | HR 0.65 (0.55-0.78) |
| Yang ^47^ | 2008 | GPRD comprising 700 General practices in UK. ≥50yrs and ≥5yrs CRC-free f/u in database. 1987-2002 | 4432 cases;44292 controls | 54.5:44.2 | Min 5 yrs use | ≥5 yrs of use  ≥10 yrs of use | Atorvastatin; Cerivastatin; Fluvastatin; Lovastatin; Pravastatin; Simvastatin | NA | 1.1 (0.5-2.2)  1.3 (0.6-2.7) |
| Shadman ^48^ | 2009 | Wisconsin Ca registry 1999-2001 & matched with community controls (licensed drivers & medicare beneficiaries) | 657 cases: 1342 controls | 0:0 | NK | ≥1prescription  <3yrs use  ≥3yrs use | Fluvastatin; Lovastatin; Pravastatin; Simvastatin | NA | 1.17 (0.74-1.85)  1.07 (0.56-2.03)  1.27 (0.68-2.38) |
| Haukka ^49^ | 2010 | National Finnish database1996-2005 (all statin users with no dx of Ca matched with one non-user) – total 944962 | 5016 cases: 939946 controls | NK | NK | ≥1prescription | Atorvastatin; Cerivastatin; Fluvastatin; Lovastatin; Pravastatin; Rosuvastatin; Simvastatin | RR 0.98 (0.98-0.99) | Colon: RR 1.01 (0.98-1.04)  Rectum RR 0.96 (0.92-0.99) |

Supplementary material 5. Flow diagram of recruitment and participation

6678 incident cases of adenocarcinoma

10,593 population based controls.

52% agree to participate (n=3471)

39% agree to participate (n=4134)

68% completed the questionnaire (n=2308)

88% completed the questionnaire (n=2974)

Resident in Tayside (n=309)

Resident in Tayside (n=294)

54 withdrawn from study (ineligible, withdrew consent, duplicate cases (n=3417)

737 withdrawn from study (ineligible, withdrew consent) (n=3396)

21,175 incident cases of adenocarcinoma

Supplementaty material 6. General description of AJCC:

The AJCC system is based on the TNM classification. In TNM classification, T stands for tumour and describes the extent of the tumour spread through the layers that form the bowel wall, N stands for nodes and indicates whether or not the cancer has spread to nearby lymph nodes and, if so, how many lymph nodes are affected and M stands for metastasis and indicates whether or not the cancer has spread to distant organs. Each of these three elements is categorised separately and classified with a number. There are five stages for tumour describing its extent through the bowel wall (Tis, T1-T4): 1) Tis, where tumour involves only the mucosa; 2) T1, where tumour invades submucosa; 3) T2, where tumour invades muscularis propria; 4) T3, where tumour invades through the muscularis propria into the subserosa, or into the pericolic or perirectal tissues; 5) T4, where tumour directly invades other organs or structures, and/or perforates. There are three stages for node describing the cancer spread to nearby lymph nodes (N0-N2): 1) N0, where there is no spread in regional lymph node; 2) N1, where there is spread in one to three regional lymph nodes; 3) N2, where there is spread in four or more regional lymph nodes. Finally, there are two stages for metastasis describing the cancer spread to distant organs (M0-M1): 1) M0, where there is no distant metastasis; 2) M1, where distant metastasis is present. In case of incomplete information regarding the tumour invasion, nodes affected and presence or not of metastasis, the stage code becomes Tx, Nx or Mx, respectively.

When the three TNM numbers are combined (stage grouping), the AJCC stage is formed (0, I-IV): 1) Stage 0 for Tis, N0 and M0; 2) Stage I for T1, N0 and M0 or T2, N0 and M0; 3) Stage IIA for T3, N0 and M0; 4) Stage IIB for T4, N0 and M0; 5) Stage IIIA for T1, N1 and M0 or T2, N1 and M0; 6) Stage IIIB for T3, N1 and M0 or T4, N1 and M0; 7) Stage IIIC for any T, N2 and M0; 8) Stage IV for any T, any N and M1; (information taken from the American cancer society;[http://www.cancer.org/](https://web.nhs.net/owa/redir.aspx?C=c1dc5beeaced465aae90c71434cb9aa2&URL=http%3A%2F%2Fwww.cancer.org%2F)).

What we did in SOCCS:

During the recruitment period Duke’s stage was recorded to describe the extent of the cancer in the body. In addition, by using Duke’s stage information we formed the AJCC stage for each case. However, for 2,719 cases metastasis information was missing and data were requested from the Scottish regional cancer networks (SCAN, WoSCAN and NoSCAN). These data were also incomplete and therefore CT scans for all patients from the Lothian region were requested (n= 578) and individually checked for evidence of metastasis. For the WoSCAN and NoSCAN regions, the consultants of individual patients were contacted by letter requesting the staging information for their patients. Following this first round of letter to consultant surgeons, it became clear that there were inconsistencies between the staging provided by the regional databases and the death status (e.g. patients noted to have metastasis in the databases were alive several years later). A second round of letters was then sent to consultant surgeons requesting clarification of metastases status of their patients. For the remaining cases with outstanding metastasis status, individual GPs were contacted by letter. This process led to only 126 cases left without staging

Supplementary material 7. Mechanism of data linkage via the Health Informatics Centre

Paper prescription - ID

SOCCS study data - ID

Drug data - CHI

SOCCS study data - CHI

Drug data, SOCCS study data

Drug data, SOCCS study data CHI

Enter data, find CHI

Find CHI via ISD

Analysis

Link using CHI

Delete CHI

**NHS**

**University**

**Health Informatics centre**

**University**

Supplementary material 8

Distribution of cases across sex, age, and health board area of residence for participants, non-participants and withdrawn subjects

| **Cases** | **Participants[[1]](#endnote-2)**  **(P) (n=3417)** | **Non-participants[[2]](#endnote-3)**  **(NP) (n=3207)** | **Withdrawn cases**  **(W) (n=54)** | **p-value**  **P vs. NP** | **p-value**  **P vs. W** |
| --- | --- | --- | --- | --- | --- |
| ***Sex*** |  |  |  |  |  |
| Men | 1958 (57.3%) | 1858 (57.9%) | 31 (57.4%) |  |  |
| Women | 1459 (42.7%) | 1342 (41.8%) | 23 (42.6%) |  |  |
| Not recorded | 0 (0.0%) | 7 (0.2%) | 0 (0.0%) | 0.02 | 0.99 |
| ***Age*** |  |  |  |  |  |
| Mean (SD) | 59.9 (11.6) | 67.0 (9.8) [[3]](#endnote-4) | 60.6 (12.4)[[4]](#endnote-5) | <5x10^-5^ | 0.67 |
| ***Health board area*** |  |  |  |  |  |
| Argyll & Clyde | 249 (7.3%) | 199 (6.2%) | 2 (3.7%) |  |  |
| Ayrshire & Arran | 228(6.7%) | 239 (7.5%) | 3 (5.6%) |  |  |
| Borders | 97 (2.8%) | 81 (2.5%) | 1 (1.8%) |  |  |
| Dumfries & Galloway | 102 (3.0%) | 106 (3.3%) | 0 (0.0%) |  |  |
| Fife | 220 (6.4%) | 180 (5.6%) | 5 (9.3%) |  |  |
| Forth Valley | 187 (5.5%) | 154 (4.8%) | 4 (7.4%) |  |  |
| Grampian | 497 (14.5%) | 282 (8.8%) | 13 (24.1%) |  |  |
| Greater Glasgow | 520 (15.2%) | 746 (23.3%) | 7 (13.0%) |  |  |
| Highland | 165 (4.8%) | 116 (3.6%) | 3 (5.6%) |  |  |
| Lanarkshire | 315 (9.2%) | 350 (10.9%) | 2 (3.7%) |  |  |
| Lothian | 533 (15.6%) | 447 (13.9%) | 7 (13.0%) |  |  |
| Orkney | 11 (0.3%) | 4 (0.1%) | 0 (0.0%) |  |  |
| Shetland | 16 (0.5%) | 9 (0.3%) | 0 (0.0%) |  |  |
| Tayside | 263 (7.7%) | 281 (8.8%) | 2 (3.7%) |  |  |
| Western Isles | 12 (0.3%) | 8 (0.3%) | 1 (1.8%) |  |  |
| Not recorded | 2 (0.1%)[[5]](#endnote-6) | 5 (0.2%) | 4 (7.4%) | <0.0005 | <0.0005 |

^*^ Agreed to participate

**[[6]](#endnote-7)** Did not agree to participate

[[7]](#endnote-8) Missing data for 56 non-participants

^§^ Missing data for 3 withdrawn participants

** Move to England

Supplementary material 9

Reason of no response for non-participants

| **Type of “no” response** | **Cases (non-participants: n=3207)** |
| --- | --- |
| Unable to take part[[8]](#endnote-9) | 1276 (39.8%) |
| Did not want to take part | 1877 (58.5%) |
| Not recorded | 54 (1.7%) |
|  |  |

^*^ Reasons for being unable to take part: deceased (n=377), exact reason not recorded (n=289), patient too ill to participate (n=276), advanced disease (n=52), unaware of diagnosis (n=33), dementia (n=29), learning difficulties (n=28), not appropriate (n=26), limited understanding (n=18), consultant not agreed for patient to be approached (n=18), patient confused (n=18), mental health problems (n=17), not approached (n=8), unable to give informed consent (n=7), communication problems (n=7), Alzheimer’s disease/ Parkinson’s disease/ Schizophrenia (n=7), unconfirmed diagnosis (n=6), patient too anxious (n=6), memory problems (n=5), patient did not speak English (n=5), patient depressed (n=3), patient did not live in Scotland (n=3), other reason (n=38).

Supplementary material 10

Distribution of controls across sex, age, health board area of residence and Carstairs deprivation index for participants, non-participants and withdrawn subjects

| **Controls** | **Participants^a^**  **(P) (n=3396)** | **Non-participants^b^**  **(NP) (n=7291)** | **Withdrawn controls**  **(W) (n=737)** | **p-value**  **P vs. NP** | **p-value**  **P vs. W** |
| --- | --- | --- | --- | --- | --- |
| ***Sex*** ^c^ |  |  |  |  |  |
| Men | 1908 (56.2%) | 4194 (57.52%) | 410 (55.63%) |  |  |
| Women | 1488 (43.8%) | 3088 (42.35%) | 327 (44.37%) |  |  |
| Not recorded | 0 (0.0%) | 9 (0.12%) | 0 (%) | 0.05^d^ | 0.78 |
| ***Age*** ^c^ |  |  |  |  |  |
| Mean (SD) | 61.2 (10.9)^e^ | 63.26 (11.43)^f^ | 63.23 (11.30)^g^ | <5x10^-5^ | <5x10^-5^ |
| ***Health board area*** ^c^ |  |  |  |  |  |
| Argyll & Clyde | 224 (6.6%) | 615 (8.4%) | 57 (7.7%) |  |  |
| Ayrshire & Arran | 233 (6.9%) | 616 (8.4%) | 37 (5.0%) |  |  |
| Borders | 111 (3.3%) | 177 (2.4%) | 23 (3.1%) |  |  |
| Dumfries & Galloway | 132 (3.9%) | 245 (3.4%) | 28 (3.8%) |  |  |
| Fife | 236 (6.9%) | 354 (4.8%) | 52 (7.1%) |  |  |
| Forth Valley | 187 (5.5%) | 373 (5.1%) | 59 (8.0%) |  |  |
| Grampian | 540 (15.9%) | 780 (10.7%) | 111 (15.1%) |  |  |
| Greater Glasgow | 416 (12.2%) | 1496 (20.1%) | 92 (12.5%) |  |  |
| Highland | 195 (5.7%) | 257 (3.5%) | 34 (4.6%) |  |  |
| Lanarkshire | 255 (7.5%) | 829 (11.4%) | 59 (8.0%) |  |  |
| Lothian | 568 (16.7%) | 956 (13.1%) | 84 (11.4%) |  |  |
| Orkney | 14 (0.4%) | 17 (0.2%) | 4 (0.5%) |  |  |
| Shetland | 13 (0.4%) | 21 (0.3%) | 8 (1.1%) |  |  |
| Tayside | 264 (7.8%) | 537 (7.4%) | 79 (10.7%) |  |  |
| Western Isles | 8 (0.2%) | 36 (0.5%) | 10 (10.7%) |  |  |
| Not recorded | 0 (0.0%) | 9 (0.1%) | 0 (0.0%) | <0.0005^h^ | <0.0005 |
| ***Carstairs deprivation index*** |  |  |  |  |  |
| 1 | 318 (9.4%) | 270 (3.7%) | 52 (7.1%) |  |  |
| 2 | 686 (20.2%) | 675 (9.3%) | 128 (17.4%) |  |  |
| 3 | 923 (27.2%) | 1086 (14.9%) | 186 (25.2%) |  |  |
| 4 | 794 (23.4%) | 1310 (18.0%) | 183 (24.8%) |  |  |
| 5 | 365 (10.7%) | 714 (9.8%) | 99 (13.4%) |  |  |
| 6 | 218 (6.4%) | 547 (7.5%) | 61 (8.3%) |  |  |
| 7 | 92 (2.7%) | 341 (4.7%) | 28 (3.8%) |  |  |
| Not recorded | 0 (0.0%) | 2348 (32.2%) | 0 (0.0%) | <0.0005***^i^*** | 0.01 |

^a^ Agreed to participate

^b^ Did not agree to participate

^c^ Sex, age and Health Board information for non-participants population controls was obtained from the cases the non-participant population controls were matched to.

^d^ The chi-square test p-value was 0.17, when we compared men and women distributions (participants versus non-participants) ignoring the 9 subjects, whose sex was not recorded.

^e^ For 17 participants, age was calculated based on the date that the PSD report was returned to the study office and for 4 participants age could not be calculated.

^f^ Age is missing for 9 non-participants population controls.

^g^ For 467 withdrawn population controls, age was calculated based on the date that the PSD report was returned to the study office and for 4 withdrawn population controls age could not be calculated.

^h^ The chi-square test p-value was <0.0005, when we compared Health Board distributions (participants versus non-participants) ignoring the 9 subjects, whose health board information was not recorded.

^i^ The chi-square p-value was <0.0005 when we compared Carstairs Deprivation Index distributions (participants versus non-participants) ignoring the 2348 subjects, whose post code sector information was either not recorded or inadequate.

**Supplementary material 11 Carstairs Deprivation Index criteria**

| **Criterion** | **Description** |
| --- | --- |
| Overcrowding | Persons in private household living at a density  of >1 person per room of all persons in private  households |
| Male unemployment | Proportion of economically active males who are  seeking work |
| Low social class | Proportion of all persons in private households  with head of household in social class 4 or 5 |
| No car | Proportion of all persons in private households  with no car |

Supplementary material 12. Characteristics of statin users versus non-users among control patients

| **Variable** | **No statin use* (n=250)** | **Statin use* (n=44)** | **P-value†** |
| --- | --- | --- | --- |
| Age at recruitment | 61.54 (11.01) | 65.7 (7.4) | 0.017 |
| Sex  Men  Women | 130 (52.0)  120 (48.0) | 31 (70.5)  13 (29.5) | 0.026 |
| FH Cancer risk  Low  Mdm/High | 223 (100)  0 (0) | 42 (97.7)  1 (2.3) | n/a |
| Smoking status  Never  Former  Current  Not known | 79 (31.6)  92 (36.8)  37 (14.8)  42 (16.8) | 11 (25.0)  23 (52.3)  8 (18.2)  2 (4.5) | 0.28 |
| Physical activity (Cycling & other sport in hours/weel) ‡  0  <3.5  3.5-7.0  >7.0 | 105 (42.0)  52 (26.2)  23 (11.6)  18 (9.1) | 23 (56.1)  8 (19.2)  6 (14.4)  4 (9.6) | 0.81 |
| BMI‡  <25  25-29.9  >30 | 68 (27.2)  92 (44.4)  47 (22.7) | 12 (28.6)  13 (31.0)  17 (40.5) | 0.021 |
| Alcohol intake (g/day) ‡ | 12.9 (14.0) | 12.4 (13.3) | 0.42 |
| Energy intake ((kJ/day) ‡ | 11235 (5064) | 10175 (2643) | 0.27 |
| DEPCAT†† ‡  1  2  3  4  5  6  7 | 27 (10.8)  57 (22.8)  67 (26.8)  57 (22.8)  20 (8.0)  21 (8.4)  1 (0.4) | 6 (13.6)  9 (20.5)  12 (27.3)  9 (20.5)  2 (4.5)  6 (13.6)  0 | 0.95 |
| PMH Bowel disease (incl IBS) | 19 (9.2) | 4 (9.8) | 0.92 |
| PMH Cancer | 10 (4.8) | 3 (7.1) | 0.53 |
| Regular use of NSAIDs**  Yes  No  Not known | 61 (24.4)  21 (8.4)  168 (67.2) | 26 (59.1)  1 (2.3)  17 (38.6) | 0.052 |
| HRT use  Yes  No  Not known | 35 (14.0)  61 (24.4)  154 (61.6) | 5 (11.4)  7 (15.9)  32 (72.7) | 0.73 |
| Hormonal contraception use  Yes  No  Not known | 36 (14.4)  59 (23.6)  155 (62.0) | 4 (9.1)  8 (18.2)  32 (72.7) | 0.76 |

* Mean values and in parenthese standard deviations for quantitative variables; number of subjects and in parentheses percentages for categorical variables

† P-values from the Pearson χ^2^ for categorical variables; from t-test for continuous variables. All statistical tests were 2-sided

‡ OR, 95% CI and P-value were computed from the logarithmic transformed variable

** Regular use = at least four times a week for at least one month

†† DEPCAT (Carstairs deprivation index) based on the 2001 Census data; 7 categories ranging from very low deprivation (DEPCAT 1) to very high deprivation (DEPCAT 7)

**Supplementary material 13. Characteristics of statin users versus non-users among female controls**

| **Variable** | **No statin use***  **(n=120)** | **Statin use***  **(n=13)** | **OR**  **(95% CI)** | **P-value** |
| --- | --- | --- | --- | --- |
| Age at recruitment | 61.4 (11.9) | 64.5 (6.4) | 0.98 (0.92, 1.03) | 0.35 |
| FH Cancer risk  Low  Mdm/High | 106 (100.0)  0 (0) | 12 (92.3)  1 (7.7) | n/a | n/a |
| Smoking status  Never  Former Current  Not known | 48 (40.0)  33 (27.5)  14 (11.7)  25 (20.8) | 4 (30.8)  7 (53.8)  1 (7.7)  1 7.7) | 0.81 (0.36, 1.85) | 0.62 |
| Physical activity (Cycling & other sport in hours/week) ‡  0  <3.5  3.5-7.0 >7.0 | 46 (51.1)  27 (30.0)  10 (11.1)  7 (7.8) | 8 (66.7)  1 (8.3)  3 (25.0)  0 | 0.83 (0.25, 2.72) | 0.76 |
| BMI‡ <25  25-29.9  >30 | 35 (37.6)  39 (41.9)  19 (20.4) | 2 (16.7)  2 (16.7)  8 (66.7) | 0.004 (0.0, 0.17) | 0.004 |
| Alcohol intake (g/day) ‡ | 6.86 (7.96) | 6.85 (10.23) | 1.12 (0.72, 1.73) | 0.62 |
| Energy intake ((kJ/day) ‡ | 10464 (6092) | 9431 (3067) | 1.62 (0.30, 8.67) | 0.57 |
| DEPCAT†† ‡  1 2  3 4  5  6  7 | 12 (10.0)  24 (20.0)  32 (26.7)  29 (24.2)  10 (8.3)  13 (10.8)  0 (0) | 0  4 (30.8)  4 (30.8)  1 (7.7)  1 (7.7)  3 (23.1)  0 | 0.88 (0.59, 1.29) | 0.50 |
| PMH Bowel disease (incl IBS) | 14 (14.7) | 1 (7.7) | 0.53 (0.06, 4.40) | 0.55 |
| PMH Cancer | 5 (5.3) | 2 (16.7) | 3.60 (0.62, 21.0) | 0.80 |
| Regular use of NSAIDs**  Yes No  Not known | 29 (24.2)  12 (10.0)  79 (65.8) | 6 (46.2)  0  7 (53.8) |  | 0.99 |
| HRT use  Yes No  Not known | 35 (29.2)  60 (50.0)  25 (20.8) | 5 (38.5)  7 (53.8)  1 (7.7) | 1.22 (0.36, 4.15) | 0.75 |
| Hormonal contraception use  Yes No  Not known | 36 (30.0)  58 (48.3)  26 (21.7) | 4 (30.8)  8 (61.5)  1 (7.7) | 0.81 (0.23, 2.87) | 0.74 |

* Mean values and in parenthese standard deviations for quantitative variables; number of subjects and in parentheses percentages for categorical variables

† P-values from the Pearson χ^2^ for categorical variables; from t-test for continuous variables. All statistical tests were 2-sided

‡ OR, 95% CI and P-value were computed from the logarithmic transformed variable

** Regular use = at least four times a week for at least one month

†† DEPCAT (Carstairs deprivation index) based on the 2001 Census data; 7 categories ranging from very low deprivation (DEPCAT 1) to very high deprivation (DEPCAT 7)

n/a one variable =zero therefore not possible to calculate OR

Supplementary material 14. Characteristics of statin users versus non-users among male controls

| **Variable** | **No statin use***  **(n=117)** | **Statin use***  **(n=31)** | **OR**  **(95% CI)** | **P-value** |
| --- | --- | --- | --- | --- |
| **Age at recruitment** | 61.6 (10.2) | 66.2 (7.9) | 0.95 (0.90, 0.99) | 0.023 |
| **FH Cancer risk Low**  **Mdm/High** | 117 (100.0)  0 | 30 (96.8)  1 (3.2) | n/a | 0.051 |
| **Smoking status Never**  **Former Current**  **Not known** | 31 (23.8)  59 (45.4)  23 (17.7)  17 (13.1) | 7 (22.6)  16 (51.6)  7 (22.6)  1 (3.2) | 0.86 (0.48, 1.55) | 0.62 |
| **Physical activity (hrs) 0**  **(Cycling & other sport)‡ <3.5**  **3.5-7.0 >7.0** | 59 (54.6)  25 (23.1)  13 (12.0)  11 (10.2) | 15 (51.7)  7 (24.1)  3 (10.3)  4 (13.7) | 1.32 (0.66, 2.64) | 0.43 |
| **BMI‡ <25**  **25-29.9**  **>30** | 33 (28.9)  53 (46.5)  28 (24.6) | 10 (33.3)  11 (36.7)  9 (30.0) | 0.43 (0.04, 4.58) | 0.48 |
| **Alcohol intake (g/day) ‡** | 18.0 (15.8) | 14.8 (13.8) | 1.30 (0.94, 1.81) | 0.12 |
| **Energy intake ((kJ/day) ‡** | 11868 (3946) | 10494 (2431) | 3.10 (0.75, 12.75) | 0.12 |
| **DEPCAT†† ‡**  **1**  **2 3**  **4 5**  **6 7** | 15 (11.5)  33 (25.4)  35 (26.9)  28 (21.5)  10 (7.7)  8 (6.2)  1 (0.8) | 6 (19.4)  5 (16.1)  8 (25.8)  8 (25.8)  1 (3.2)  3 (9.7)  0 | 1.02 (0.77, 1.35) | 0.90 |
| **PMH Bowel disease (incl IBS)** | 5 (4.5) | 3 (10.3) | 2.45 (0.55, 10.90) | 0.24 |
| **PMH Cancer** | 5 (4.4) | 1 (3.3) | 0.75 (0.08, 6.69) | 0.80 |
| **Regular use of NSAIDs****  **Yes No**  **Not known** | 32 (24.6)  9 (6.9)  89 (68.5) | 20 (64.5)  1 (3.2)  10 (32.3) | 5.63 (0.66, 47.82) | 0.11 |
| **HRT use**  **Yes No**  **Not known** | n/a | n/a | n/a | n/a |
| **Hormonal contraception use**  **Yes No**  **Not known** | n/a | n/a | n/a | n/a |

* Mean values and in parenthese standard deviations for quantitative variables; number of subjects and in parentheses percentages for categorical variables

† P-values from the Pearson χ^2^ for categorical variables; from t-test for continuous variables. All statistical tests were 2-sided

‡ OR, 95% CI and P-value were computed from the logarithmic transformed variable

** Regular use = at least four times a week for at least one month

†† DEPCAT (Carstairs deprivation index) based on the 2001 Census data; 7 categories ranging from very low deprivation (DEPCAT 1) to very high deprivation (DEPCAT 7)

n/a one variable =zero therefore not possible to calculate OR

Supplementary material 15 Association between statin use and use of sigmoidoscopy and/or colonoscopy

Statin category: Having been prescribed at least one prescription of statin

| Statin | Sigmoidoscopy |  |  |  |
| --- | --- | --- | --- | --- |
|  | Yes | No | Don’t know | missing |
| Yes | 22 | 110 | 0 | 7 |
| No | 37 | 301 | 4 | 122 |
| *chi2 p-value* |  |  | *0.11* | *<0.0005* |
|  | Colonoscopy |  |  |  |
| Yes | 14 | 115 | 3 | 7 |
| No | 30 | 306 | 4 | 124 |
| *chi2 p-value* |  |  | *0.55* | *<0.0005* |

Statin category: Having been prescribed more than one prescription of statin

| Statin | Sigmoidoscopy |  |  |  |
| --- | --- | --- | --- | --- |
|  | Yes | No | Don’t know | missing |
| Yes | 19 | 89 | 0 | 6 |
| No | 40 | 322 | 4 | 123 |
| *chi2 p-value* |  |  | *0.11* | *<0.0005* |
|  | Colonoscopy |  |  |  |
| Yes | 11 | 94 | 3 | 6 |
| No | 33 | 327 | 4 | 125 |
| *chi2 p-value* |  |  | *0.41* | *<0.0005* |

Statin category: Having been prescribed at least one prescription of statin with the first prescription being at least two months pre-recruitment

| Statin | Sigmoidoscopy |  |  |  |
| --- | --- | --- | --- | --- |
|  | Yes | No | Don’t know | missing |
| Yes | 10 | 55 | 0 | 4 |
| No | 49 | 356 | 4 | 125 |
| *chi2 p-value* |  |  | *0.55* | *0.005* |
|  | Colonoscopy |  |  |  |
| Yes | 6 | 57 | 2 | 4 |
| No | 38 | 364 | 5 | 127 |
| *chi2 p-value* |  |  | *0.52* | *0.004* |

Statin category: Having been prescribed at least one prescription of statin with the first prescription being at least seven months pre-recruitment

| Statin | Sigmoidoscopy |  |  |  |
| --- | --- | --- | --- | --- |
|  | Yes | No | Don’t know | missing |
| Yes | 9 | 49 | 0 | 4 |
| No | 50 | 362 | 4 | 125 |
| *chi2 p-value* |  |  | *0.58* | *0.02* |
|  | Colonoscopy |  |  |  |
| Yes | 6 | 50 | 2 | 4 |
| No | 38 | 371 | 5 | 127 |
| *chi2 p-value* |  |  | *0.39* | *0.009* |

Statin category: Having been prescribed two or more prescriptions of statin with the first prescription being at least two months pre-recruitment

| Statin | Sigmoidoscopy |  |  |  |
| --- | --- | --- | --- | --- |
|  | Yes | No | Don’t know | missing |
| Yes | 10 | 48 | 0 | 4 |
| No | 49 | 363 | 4 | 125 |
| *chi2 p-value* |  |  | *0.39* | *0.01* |
|  | Colonoscopy |  |  |  |
| Yes | 6 | 50 | 2 | 4 |
| No | 38 | 371 | 5 | 127 |
| *chi2 p-value* |  |  | *0.39* | *0.009* |

Statin category: Having been prescribed two or more prescriptions of statin with the first prescription being at least seven months pre-recruitment

| Statin | Sigmoidoscopy |  |  |  |
| --- | --- | --- | --- | --- |
|  | Yes | No | Don’t know | missing |
| Yes | 9 | 44 | 0 | 4 |
| No | 50 | 367 | 4 | 125 |
| *chi2 p-value* |  |  | *0.45* | *0.02* |
|  | Colonoscopy |  |  |  |
| Yes | 6 | 45 | 2 | 4 |
| No | 38 | 376 | 5 | 127 |
| *chi2 p-value* |  |  | *0.29* | *0.01* |

Supplementary material 16 Association between colorectal cancer and statin use among 211 cases and 194 control patients

| **Statin use** | **Cases (194)** | **Controls (211)** | **Basic OR***  **(95% CI)**  **P-value** | **Adjusted OR†**  **(95% CI)**  **P-value** |
| --- | --- | --- | --- | --- |
| No use of statins | 154 | 145 | 1.0 (referent) | 1.0 (referent) |
| ≥1dispensed prescription at least 2 months pre-recruitment | 15 | 38 | 0.40 (0.21, 0.76)  0.01** | 0.33 (0.16, 0.70)  0.004** |
| ≥1dispensed prescription at least 7 months pre-recruitment | 14 | 32 | 0.46 (0.24, 0.90)  0.02** | 0.4 (0.18, 0.86)  0.02** |
| ≥2 dispensed prescriptions at least 2 months pre-recruitment | 14 | 32 | 0.46 (0.23, 0.90)  0.02** | 0.43 (0.20, 0.94)  0.03** |
| ≥2 dispensed prescriptions at least 7 months pre-recruitment | 13 | 28 | 0.50 (0.25, 1.00)  0.05 | 0.50 (0.23, 1.10)  0.08 |

* Adjusted for matching factors (age +/-1year), sex and region of residence). OR = Odds ratio; CI = confidence interval

† Adjusted for matching factors ((age +/-1year), sex and region of residence), Family history of cancer, past medical history of cancer, past medical history of bowel disease, BMI, smoking, physical activity and regular NSAID intake

** statistically significant at p<0.05

Supplementary material 17. AJCC distribution according to statin use

| **Statin use description** | **Status** | **AJCC** | | | |
| --- | --- | --- | --- | --- | --- |
|  |  | **1** | **2** | **3** | **4** |
| Statin use: at least **1** prescription | No | 54  (80.6%) | 82  (78.9%) | 73  (81.1%) | 37  (90.2%) |
|  | Yes | 13  (19.4%) | 22  (21.1%) | 17  (18.9%) | 4  (9.8%) |
|  | *p-value* | 0.46 |  |  |  |
| Statin use: at least **1** prescription dispensed before diagnosis | No | 54  (91.5%) | 82  (91.1%) | 73  (89.0%) | 37  (90.2%) |
|  | Yes | 5  (8.5%) | 8  (8.9%) | 9  (11.0%) | 4  (9.8%) |
|  | *p-value* | 0.96 |  |  |  |
| Statin use: **2+** prescriptions | No | 56  (83.6%) | 84  (80.8%) | 76  (84.4%) | 37  (90.2%) |
|  | Yes | 11  (16.4%) | 20  (19.2%) | 14  (15.6%) | 4  (9.8%) |
|  | *p-value* | *0.58* |  |  |  |
| Statin use: **2+** prescriptions dispensed before diagnosis | No | 54  (91.5%) | 82  (91.1%) | 74  (90.2%) | 37  (90.2%) |
|  | Yes | 5  (8.5%) | 8  (8.9%) | 8  (9.8%) | 4  (9.8%) |
|  | *p-value* | *0.99* |  |  |  |

Supplementary material 18. Published meta-analyses of RCTs investigating the association between colorectal cancer risk and statin use

| **Authors** | **Year of publication** | **No. of studies included** | **Total number of participants (statin users:controls)** | **Mean follow up (yrs)** | **Colorectal Cancer incidence**  **Risk Ratio/OR (95% CI) (random effects model)** | **Comment** |
| --- | --- | --- | --- | --- | --- | --- |
| Bjerre LM et al ^50^ | 2001 | 2 | 10,764 | ≈5 | No value given | For Ca overall the findings were RR 0.999 (0.99-1.01).In considering site-specific Ca outcomes the outcome was not statistically significant. |
| Bonovas S et al ^51^ | Aug 2007 | 6 | 55,113 (27,557:27,556) | 5.9 | RR 0.95 (0.8 to 1.13) |  |
| Browning DRL Martin RM ^52^ | Nov 2006 | 6  3 | 45430  20063 | ≤5  >5 | RR 1.02 (0.87-1.19)  RR 1.02 (0.79-1.30) |  |
| Dale ^53^ | Jan 2006 | 4 | 27,972  (13,984:13,988) | ≥1 | OR 0.95  (0.73 to 1.25) | p-value 0.24 |

Supplementary material 19. Published meta-analyses of observational studies investigating the association between colorectal cancer risk and statin use

| **Authors** | **Year of publication** | **No. of studies included** | **Total number of participants (statin users:controls)** | **CRC incidence**  **RR/ OR (95% CI) (random effects model)** |
| --- | --- | --- | --- | --- |
| Browning and Martin ^52^ | 2007 | 5 | 509051 | RR 0.84 (0.59-1.21) p=0.36 |
| Bonovas et al ^54^ | 2007 | 12 | 1.5 million | RR 0.92 (0.88-0.96) |
| Taylor M et al ^55^ | 2008 | 6 | 969636 | OR 0.89 (0.82-0.97) |

Supplementary material 20. Post hoc power calculation

**All-cause mortality**

Please see below the sample size (N) and number of all cause deaths (E) that are required in order to have 80% power to detect effects (HR) that were reported in table 3 for all cause mortality for a p-value level (alpha) of 0.05 and given that the all cause death rate was 0.34 (106/308).

+---------------------------------------------------------------+

Power N E HR SD Alpha* Pr(E)

---------------------------------------------------------------

.8 89 31 .36 .5 .05 .34

.8 182 62 .49 .5 .05 .34

.8 204 70 .51 .5 .05 .34

.8 244 83 .54 .5 .05 .34

.8 293 100 .57 .5 .05 .34

.8 312 106 .58 .5 .05 .34

.8 332 113 .59 .5 .05 .34

.8 378 129 .61 .5 .05 .34

.8 433 148 .63 .5 .05 .34

.8 464 158 .64 .5 .05 .34

.8 498 170 .65 .5 .05 .34

.8 576 196 .67 .5 .05 .34

.8 726 247 .7 .5 .05 .34

.8 1227 417 .76 .5 .05 .34

.8 2660 905 .83 .5 .05 .34

.8 4762 1619 .87 .5 .05 .34

+---------------------------------------------------------------+

* two sided

**Colorectal-cancer specific mortality**

Please see below the sample size (N) and number of CRC-specific deaths (E) that are required in order to have 80% power to detect effects (HR) that were reported in table 3 for CRC-specific mortality for a p-value level (alpha) of 0.05 and given that the all cause death rate was 0.30 (91/308).

+---------------------------------------------------------------+

| Power N E HR SD Alpha* Pr(E) |

|---------------------------------------------------------------|

| .8 43 13 .21 .5 .05 .3 |

| .8 86 26 .33 .5 .05 .3 |

| .8 95 29 .35 .5 .05 .3 |

| .8 140 42 .42 .5 .05 .3 |

| .8 231 70 .51 .5 .05 .3 |

| .8 245 74 .52 .5 .05 .3 |

| .8 276 83 .54 .5 .05 .3 |

| .8 312 94 .56 .5 .05 .3 |

| .8 353 106 .58 .5 .05 .3 |

| .8 402 121 .6 .5 .05 .3 |

| .8 429 129 .61 .5 .05 .3 |

| .8 526 158 .64 .5 .05 .3 |

| .8 564 170 .65 .5 .05 .3 |

| .8 653 196 .67 .5 .05 .3 |

| .8 3443 1033 .84 .5 .05 .3 |

| .8 5397 1619 .87 .5 .05 .3 |

+---------------------------------------------------------------+

* two sided

Supplementary material 21. Quantity of missing data for cases and controls

| **Variable** | **Cases (n=309) No(%)** | **Controls (n=294)** |
| --- | --- | --- |
| Family history of CRC | 22 (7) | 28 (10) |
| Smoking history | 78 (25) | 44 (15) |
| Physical activity | 88 (28) | 55 (19) |
| BMI | 79 (26) | 45 (15) |
| Alcohol | 98 (32) | 59 (20) |
| Energy | 98 (32) | 59 (20) |
| Deprivation category | 0 (0) | 0 (0) |
| PMH Cancer | 77 (25) | 43 (15) |
| PMH Bowel disease | 79 (21) | 47 (16) |
| NSAID use | 78 (25) | 43 (15) |
| Regular NSAID use | 116 (34) | 47 (16) |

| **Variable** | **Cases (n=149)** | **Controls (n=133)** |
| --- | --- | --- |
| HRT use | 42 (28) | 25 (19) |
| Hormonal contraception use | 42 (28) | 25 (19) |

References

1. Graaf MR, Richel DJ, van Noorden CJ, Guchelaar HJ. Effects of statins and farnesyltransferase inhibitors on the development and progression of cancer. *Cancer Treatment Reviews* 2004;30(7):609-41.

2. Guruswamy S, Rao CV. Multi-target approaches in colon cancer chemoprevention based on systems biology of tumor cell-signaling. *Gene Regulation and Systems Biology* 2008(of Publication: 2008):2008(2)(pp 163-176), 08.

3. Hopfner M, Sutter AP, Huether A, Baradari V, Scherubl H. Tyrosine kinase of insulin-like growth factor receptor as target for novel treatment and prevention strategies of colorectal cancer. *World Journal of Gastroenterology* 2006;12(35):5635-43.

4. McAnally JA, Jung M, Mo HB. Farnesyl-O-acetylhydroquinone and geranyl-O-acetylhydroquinone suppress the proliferation of murine B16 melanoma cells, human prostate and colon adenocarcinoma cells, human lung carcinoma cells, and human leukemia cells. *Cancer Letters* 2003;202(2):181-92.

5. Ukomadu C, Dutta A. p21-dependent inhibition of colon cancer cell growth by mevastatin is independent of inhibition of G(1) cyclin-dependent kinases. *Journal of Biological Chemistry* 2003;278(44):43586-94.

6. Xiao H, Zhang Q, Lin Y, Reddy BS, Yang CS. Combination of atorvastatin and celecoxib synergistically induces cell cycle arrest and apoptosis in colon cancer cells. *International Journal of Cancer* 2008;122(9):2115-24.

7. Jakobisiak M, Bruno S, Skierski JS, Darzynkiewicz Z. Cell cycle-specific effects of lovastatin. . *Proceedings of the National Academy of Science* 1991;88:3628-32.

8. Keyomarsi K, Sandoval L, Band V, Pardee A. Synchronization of tumor and normal cells from G1 to multiple cell cycles by Lovastatin. . *Cancer Research* 1991;51:3602-9.

9. Gniadecki R. Depletion of membrane cholesterol causes ligand-independent activation of Fas and apoptosis. *Biochem Biophys* 2004;320:165-9.

10. Sleijfer S, van der Gaast A, Planting AST, Stoter G, Verweij J. The potential of statins as part of anti-cancer treatment. *European Journal of Cancer* 2005;41(4):516-22.

11. Agarwal B, Bhendwal S, Halmos B, Moss SF, Ramey WG, Holt PR. Lovastatin augments apoptosis induced by chemotherapeutic agents in colon cancer cells. *Clinical Cancer Research* 1999;5(8):2223-29.

12. Agarwal B, Halmos B, Feoktistov AS, et al. Mechanism of lovastatin-induced apoptosis in intestinal epithelial cells. *Carcinogenesis* 2002;23(3):521-28.

13. Cho S, Kim J, Kim J, Lee J, Jung H, Song I. Simvastatin induces apoptosis in human colon cancer cells and in tumor xenografts, and attenuates colitis-associated colon cancer in mice. *International Journal of Cancer* 2008;123:951-7.

14. Kodach LL, Bleuming SA, Peppelenbosch MP, Hommes DW, van den Brink GR, Hardwick JCH. The effect of statins in colorectal cancer is mediated through the bone morphogenetic protein pathway. *Gastroenterology* 2007;133(4):1272-81.

15. Wong WWL, Dimitroulakos J, Minden MD, Penn LZ. HMG-CoA reductase inhibitors and the malignant cell: the statin family of drugs as triggers of tumor-specific apoptosis. *Leukemia* 2002;16(4):508-19.

16. Setoguchi S, Glynn RJ, Avorn J, Mogun H, Schneeweiss S. Statins and the risk of lung, breast, and colorectal cancer in the elderly. *Circulation* 2007;115(1):27-33.

17. Dimitroulakos J, Lorimer IA, Goss G. Strategies to enhance epidermal growth factor inhibition: Targeting the mevalonate pathway. *Clinical Cancer Research* 2006;12(14):4426S-31S.

18. Dulak J, Jozkowicz A. Anti-angiogenic and anti-inflammatory effects of statins: Relevance to anti-cancer therapy. *Current Cancer Drug Targets* 2005;5(8):579-94.

19. Dulak J, Loboda A, Jazwa A, et al. Atorvastatin affects several angiogenic meditors in human endothelial cells. *Endothilium* 2005;12(5-6):233-41.

20. Aarons C, Reed KL, Bajenova O, Thomas P, Becker JM, Stucchi AF. Atorvastatin reduces the attachment of colorectal cancer cells to endothelial cells in vitro by Down-regulating the endothelial cell surface expression of adhesion molecules. *Gastroenterology* 2006;130(4):A679-A79.

21. Asakage M, Tsuno NH, Kitayama J, et al. 3-Hydroxy-3-methylglutaryl-coenzyme A reductase inhibitor (pravastatin) inhibits endothelial cell proliferation dependent on G(1) cell cycle arrest. *Anti-Cancer Drugs* 2004;15(6):625-32.

22. Vincent L, Chen W, Hong L, et al. Inhibition of endothelial cell migration by cerivastatin, an HMG-CoA reductase inhibitor: contribution to its anti-angiogenic effect. *Febs Letters* 2001;495(3):159-66.

23. Chan KKW, Oza AM, Siu LL. The Statins as Anticancer Agents. *Clin Cancer Res* 2003;9(1):10-19.

24. Nubel T, Dippold W, Kleinert H, Kaina B, Fritz G. Lovastatin inhibits Rho-regulated expression of E-selectin by TNFalpha and attenuates tumor cell adhesion. *Faseb Journal* 2004;18:140-42.

25. Agarwal B, Rao CV, Bhendwal S, et al. Lovastatin augments sulindac-induced apoptosis in colon cancer cells and potentiates chemopreventive effects of sulindac. *Gastroenterology* 1999;117(4):838-47.

26. Feleszko W, Jalili A, Olszewska D, et al. Synergistic interaction between highly specific cyclooxygenase-2 inhibitor, MF-tricyclic and lovastatin in murine colorectal cancer cell lines. *Oncology Reports* 2002;9(4):879-85.

27. Feleszko W, Mlynarczuk I, Balkowiec-Iskra EZ, et al. Lovastatin potentiates antitumor activity and attenuates cardiotoxicity of doxorubicin in three tumor models in mice. *Clinical Cancer Research* 2000;6(5):2044-52.

28. Guruswamy S, Rao CV. Synergistic effects of lovastatin and celecoxib on caveolin-1 and its down-stream signaling molecules: Implications for colon cancer prevention. *International Journal of Oncology* 2009(of Publication: November 2009):35(5)(pp 1037-43), 2009.

29. Jin Z, Dicker D, El-Deiry W. Enhanced sensitivity of G1 arrested human cancer cells suggests a novel therapeutic strategy using a combination of simvastatin and TRAIL, Cell Cycle 1 (2002), pp. 82–89. *Cell Cycle* 2002;1:82-9.

30. Lee J, Jung KH, Park YS, et al. Simvastatin plus irinotecan, 5-fluorouracil, and leucovorin (FOLFIRI) as first-line chemotherapy in metastatic colorectal patients: a multicenter phase II study. *Cancer Chemotherapy & Pharmacology* 2009;64(4):657-63.

31. Wachtershauser A, Akoglu B, Stein J. HMG-CoA reductase inhibitor mevastatin enhances the growth inhibitory effect of butyrate in the colorectal carcinoma cell line Caco-2. *Carcinogenesis* 2001;22(7):1061-67.

32. Feleszko W, Zagozdzon R, Gołab J, Jakóbisiak M. Potentiated antitumour effects of cisplatin and lovastatin against MmB16 melanoma in mice." European Journal of Cancer 34(3): 406-411. *European Journal of Cancer* 1998;34(3):406-11.

33. Yang Y, Hennessy S, Propert K, Hwang W, Sarkar M, Lewis J. Chronic statin therapy and the risk of colorectal cancer *Pharmacoepidemiology and Drug Safety* 2008;17(9):869-76.

34. Friis S, Poulsen AH, Johnsen SP, et al. Cancer risk among statin users: A population-based cohort study. *International Journal of Cancer* 2005;114(4):643-47.

35. Jacobs EJ, Rodriguez C, Brady KA, Connell CJ, Thun MJ, Calle EE. Cholesterol-lowering drugs and colorectal cancer incidence in a large United States Cohort. *Journal of the National Cancer Institute* 2006;98(1):69-72.

36. Flick E, Habel L, Chan K, et al. Statin use and risk of colorectal cancer in a cohort of middle-aged men in the US: a prospective cohort study. *Drugs* 2009;69(11):1445-57.

37. Singh H, Mahmud S, Turner D, Xue L, Demers A, Bernstein C. Long-term use of statins and risk of colorectal cancer: a population-based study. *American Journal of Gastroenterology*  2009;104(12):3015-23.

38. Blais L, Desgagne A, LeLorier J. 3-Hydroxy-3-methylglutaryl coenzyme A reductase inhibitors and the risk of cancer: a nested case-control study.[see comment]. *Archives of Internal Medicine* 2000;160:2363-68.

39. Graaf MR, Beiderbeck AB, Egberts ACG, Richel DJ, Guchelaar HJ. The risk of cancer in users of statins. *Journal of Clinical Oncology* 2004;22(12):2388-94.

40. Kaye JA, Jick H. Statin use and cancer risk in the General Practice Research Database. *British Journal of Cancer* 2004;90(3):635-37.

41. Poynter JN, Gruber SB, Higgins PDR, et al. Statins and the risk of colorectal cancer. *New England Journal of Medicine* 2005;352(21):2184-92.

42. Coogan P, Smith J, Rosenberg L. Statin use and the risk of colorectal cancer. *Journal of the National Cancer Institute* 2007;99:32-40.

43. Coogan PF, Rosenberg L, Strom BL. Statin use and the risk of 10 cancers. *Epidemiology* 2007;18(2):213-19.

44. Vinogradova Y, Hippisley-Cox J, Coupland C, Logan R. Risk of colorectal cancer in patients prescribed statins, nonsteroidal anti-inflammatory drugs, and cyclooxygenase-2 inhibitors: nested case-control study. *Gastroenterology* 2007;133(2):393-402.

45. Hoffmeister M, Chang-Claude J, Brenner H. Individual and joint use of statins and low-dose aspirin and risk of colorectal cancer: a population-based case-control study. *International Journal of Cancer* 2007;121(6):1325-30.

46. Farwell W, Scranton R, Lawler E, et al. The association between statins and cancer incidence in a veterans population. *Journal of the National Cancer Institute* 2008;100(2):134-9.

47. Yang Y, Hennessy S, Propert K, Hwang W, Sarkar M, Lewis J. Chronic statin therapy and the risk of colorectal cancer. *Pharmacoepidemiology and Drug Safety* 2008;17(9):869-76.

48. Shadman M, Newcomb P, Hampton J, Wernli K, Trentham-Dietz A. Non-steroidal anti-inflammatory drugs and statins in relation to colorectal cancer risk. *World Journal of Gastroenterology* 2009;15(19):2336-9.

49. Haukka J, Sankila R, Klaukka T, et al. Incidence of cancer and statin usage--record linkage study. *International Journal of Cancer* 2010;126(1):279-84.

50. Bjerre LM, LeLorier J. Do statins cause cancer? A meta-analysis of large randomized clinical trials. *American Journal of Medicine* 2001;110(9):716-23.

51. Bonovas S, Sitaras NM. Does pravastatin promote cancer in elderly patients? A meta-analysis. *Canadian Medical Association Journal* 2007;176(5):649-54.

52. Browning D, Martin R. Statins and risk of cancer: A systematic review and metaanalysis. *International Journal of Cancer* 2007;120(4):833-43.

53. Dale KM, Coleman CI, Henyan NN, Kluger J, White CM. Statins and cancer risk - A meta-analysis. *Jama-Journal of the American Medical Association* 2006;295(1):74-80.

54. Bonovas S, Filioussi K, Tsavaris N, Sitaras NM. Statins and cancer risk: A literature-based meta-analysis and meta-regression analysis of 35 randomized controlled trials. *Journal of Clinical Oncology* 2006;24(30):4808-17.

55. Taylor M, Wells B, Smolak M. Statins and cancer: a meta-analysis of case-control studies. *European Journal of Cancer Prevention* 2008;17:259-68.

1. Agreed to participate [↑](#endnote-ref-2)
2. Did not agree to participate [↑](#endnote-ref-3)
3. Missing data for 56 non-participants [↑](#endnote-ref-4)
4. Missing data for 3 withdrawn participants [↑](#endnote-ref-5)
5. Move to England [↑](#endnote-ref-6)
6. Did not agree to participate [↑](#endnote-ref-7)
7. Missing data for 56 non-participants [↑](#endnote-ref-8)
8. Reasons for being unable to take part: deceased (n=377), exact reason not recorded (n=289), patient too ill to participate (n=276), advanced disease (n=52), unaware of diagnosis (n=33), dementia (n=29), learning difficulties (n=28), not appropriate (n=26), limited understanding (n=18), consultant not agreed for patient to be approached (n=18), patient confused (n=18), mental health problems (n=17), not approached (n=8), unable to give informed consent (n=7), communication problems (n=7), Alzheimer’s disease/ Parkinson’s disease/ Schizophrenia (n=7), unconfirmed diagnosis (n=6), patient too anxious (n=6), memory problems (n=5), patient did not speak English (n=5), patient depressed (n=3), patient did not live in Scotland (n=3), other reason (n=38). [↑](#endnote-ref-9)
